# Grey-matter network disintegration as predictor of cognitive and motor function with aging

**DOI:** 10.1007/s00429-018-1642-0

**Published:** 2018-03-06

**Authors:** Marisa Koini, Marco Duering, Benno G. Gesierich, Serge A. R. B. Rombouts, Stefan Ropele, Fabian Wagner, Christian Enzinger, Reinhold Schmidt

**Affiliations:** 1 Division of Neurogeriatrics, Department of Neurology, Medical University of Graz, Auenbruggerplatz 22, 8036 Graz, Austria; 2 Institute for Stroke and Dementia Research, Ludwig-Maximilians-University, Munich, Germany; 3 Department of Radiology, Leiden University Medical Center, Leiden, The Netherlands; 4 Institute of Psychology, Leiden University, Leiden, The Netherlands

**Keywords:** Structural covariance networks, Grey-matter atrophy, Cognition, Fine motor skills, Aging

## Abstract

Loss of grey-matter volume with advancing age affects the entire cortex. It has been suggested that atrophy occurs in a network-dependent manner with advancing age rather than in independent brain areas. The relationship between networks of structural covariance (SCN) disintegration and cognitive functioning during normal aging is not fully explored. We, therefore, aimed to (1) identify networks that lose GM integrity with advancing age, (2) investigate if age-related impairment of integrity in GM networks associates with cognitive function and decreasing fine motor skills (FMS), and (3) examine if GM disintegration is a mediator between age and cognition and FMS. T1-weighted scans of *n* = 257 participants (age range: 20–87) were used to identify GM networks using independent component analysis. Random forest analysis was implemented to examine the importance of network integrity as predictors of memory, executive functions, and FMS. The associations between GM disintegration, age and cognitive performance, and FMS were assessed using mediation analyses. Advancing age was associated with decreasing cognitive performance and FMS. Fourteen of 20 GM networks showed integrity changes with advancing age. Next to age and education, eight networks (fronto-parietal, fronto-occipital, temporal, limbic, secondary somatosensory, cuneal, sensorimotor network, and a cerebellar network) showed an association with cognition and FMS (up to 15.08%). GM networks partially mediated the effect between age and cognition and age and FMS. We confirm an age-related decline in cognitive functioning and FMS in non-demented community-dwelling subjects and showed that aging selectively affects the integrity of GM networks. The negative effect of age on cognition and FMS is associated with distinct GM networks and is partly mediated by their disintegration.

## Introduction

Aging is associated with widespread cerebral grey-matter atrophy (Sigurdsson et al. 2012). Age-related loss of grey matter is more pronounced in men (up to − 0.70% per year) than in women (up to − 0.55% per year) (Alexander et al. 2005; Enzinger et al. 2005; Sigurdsson et al. 2012) and differs among brain regions (Fjell and Walhovd 2010). Hence, global measures of brain atrophy do not depict areas of increased vulnerability due to aging and disregard the notion of the brain being organized in networks maturing and dying together (Alexander-Bloch et al. 2013). Age-related atrophy is most pronounced in the hippocampus, caudate nucleus, association cortex, cerebellum, and the medial temporal lobe, while little volume loss is seen in other cortical regions such as the entorhinal cortex and the primary visual cortex (Salat et al. 2004; Alexander et al. 2005; Raz et al. 2005; Zielinski et al. 2010; Jiang et al. 2014). It has been recognized using cortical thickness networks that intra-individual differences in the structure of a brain region covary with the structure of other brain regions (structural covariance, SCN) (Lerch et al. 2006).

DuPre and Spreng (2017) showed that age-related disintegration follows these structural covariance networks. Indeed, studies examining the structural covariance have shown that disintegration in several canonical networks, such as the default mode network, the dorsal attention network, the fronto-parietal network, the somatomotor network, the ventral attention network, or a language-related semantic network are strongly associated with age, while others, such as a temporal network, an auditory network or cerebellar networks remained relatively preserved during the aging process (Montembeault et al. 2012; Hafkemeijer et al. 2014; Foster-Dingley et al. 2016; DuPre and Spreng 2017). A network involving areas for transmodal processing, including the lateral prefrontal cortex, the frontal eye field, the intraparietal cortex, the superior temporal sulcus, the posterior cingulate cortex, and the medial temporal lobe, has shown an inverted u-shaped age trajectory and vulnerability in different pathologies (i.e., Alzheimer’s disease and schizophrenia) and an association to memory performance (Douaud et al. 2014). Hence, a grey-matter network-dependent vulnerability creates a biological basis for cognitive deterioration during aging (Seeley et al. 2009).

Considering the association with cognition, the previous studies indeed found impaired age-related structural covariance to be associated with cognitive dysfunction (Brickman et al. 2007; Steffener et al. 2013; Spreng and Turner 2013; Douaud et al. 2014) and fine motor skills (Hoogendam et al. 2014). We here extend the previous work by examining network disintegration and its association with cognitive functioning and fine motor skills over a wide age range in a large community-dwelling cohort.

We identified those grey-matter SCNs that are affected by aging, determined their domain-specific association with cognitive function, and assessed if associations between age and cognitive function and fine motor skills are mediated by grey-matter network disintegration.

## Methods

### Compliance with ethical standards

The study was approved by the ethics committee of the Medical University of Graz, Austria. Informed consent was obtained from all individual participants included in the study. Disclosures of authors are quoted at the end of the manuscript.

### Subjects and assessments

**Fig. 1:**
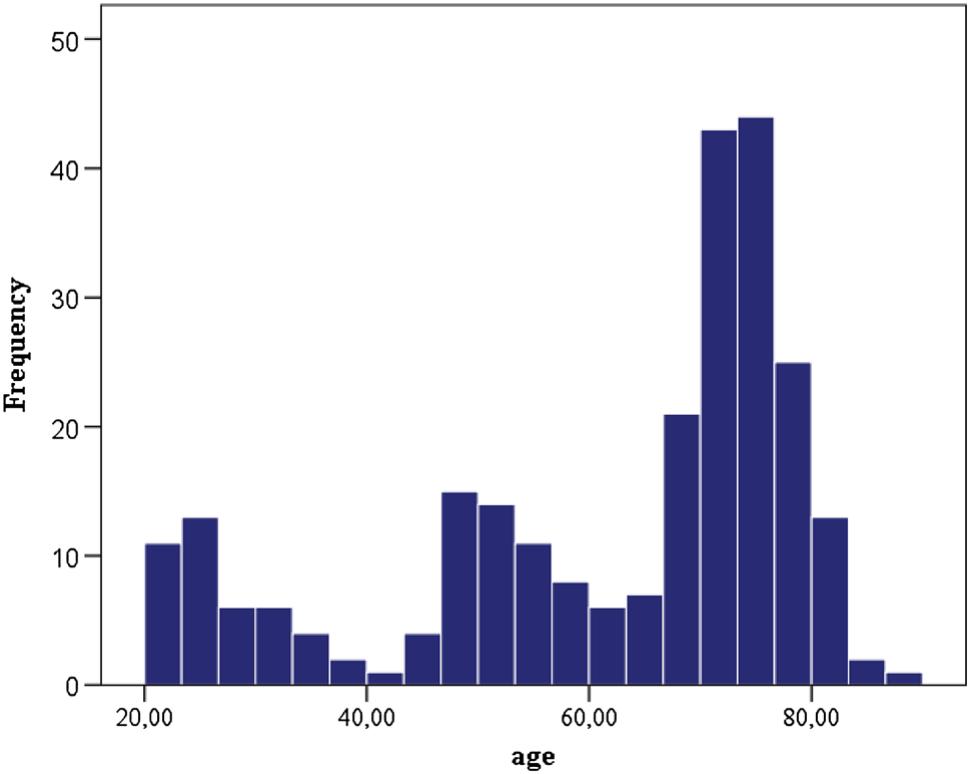
Age distribution of the total study sample (*n* = 257)

Overall, 257 non-demented subjects were included in the study. Two-hundred sixteen of them were elderly participants of the Austrian Stroke Prevention Study (ASPS), with a mean age of 68.2 ± 10.7 years, and 41 were younger individuals who have been invited to serve as healthy participants in a different study with a mean age of 26.9 ± 4.7 years. In total, there were 113 men and 144 women. All subjects underwent an identical imaging protocol. The 41 young subjects were included to broaden the age range of the ASPS from 39 to 87 years for the age stratum of 20–38 years (for age distribution, see Fig. 1). The ASPS is a single-center, prospective, follow-up study on the cerebral effects of vascular and genetic risk factors in the normal elderly population of Graz, Austria (Schmidt et al. 2003, 2005). Individuals were excluded from the ASPS study if they had a history of neuropsychiatric disease, including cerebrovascular attacks and dementia, or an abnormal neurologic examination determined on the basis of a structured clinical interview and physical and neurologic examinations. The same selection criteria were applied to the 41 subjects that were recruited to extend the range of the study down to 20 years of age.

All ASPS participants, but not the 41 younger subjects, underwent neuropsychological testing including tests on memory, executive functions, and fine motor skills. The tests employed have been widely used in the German speaking area and were always applied in the same order and under unchanged laboratory conditions. Intermediate memory recall and learning ability was assessed by the “Bäumler’s Lern- und Gedächtnistest” (LGT-3) (Bäumler 1974), a highly demanding paper–pencil procedure consisting of six subtests. Three subtests (word and digit association tasks, and story recall) screen for verbal memory, and two subtests (trail and design recall) screen for figural memory. The sum of weighted scores from these subtests and of an image recognition paradigm results in the total learning and memory performance score. The subtests were weighted based on the reliability scores provided in the test manual. The stimulus sets of the word association task (German–Turkish word pairs), the story (facts about construction of a library), and design recall (core symbol and frame), and the recognition paradigm (objects) consist of 20 items each. A trail in an abstracted city map serves as the trail recall test. These sets of stimuli were presented to the person being tested for 1 min. Two minutes were given for learning the 13 items of the digit association task (three-digit telephone numbers and names of extension holders). During a learning phase, the six sets of stimuli are subsequently presented to the person being tested. The recall phase starts immediately thereafter and follows the same order. The delay between presentation and recall for a given subtest ranges between 7 and 11 min. Executive functions were tested by the part B of the trail making test (Department 1944) and the digit span forward and backwards, which is part of the Wechsler adult intelligence scale, revised (Tewes 1991). Fine motor skills were evaluated by the Purdue pegboard test (Tiffin and Asher 1948). All scores were *z*-transformed for harmonization. The Mini Mental State Examination (MMSE) was used for the evaluation of the presence of Mild Cognitive Impairment (MCI). A subject was classified as mild cognitively impaired when the subject’s test score was below the 25th percentile of age and education specific norm data (Crum et al. 1993).

### Image acquisition

Magnetic resonance imaging was performed on a 3T whole body scanner (TimTrio; Siemens Healthcare, Erlangen, Germany) with a 12-channel head coil. For each participant, a high-resolution T1-weighted three-dimensional anatomical image with magnetization preparation (MPRAGE) and whole brain coverage (TR = 1900 ms, TE = 2.19 ms, inversion time = 900 ms, flip angle = 9°, isotropic resolution of 1 mm) was acquired. For the identification of white matter hyperintensities, an axial T2-weighted FLAIR sequence (TR = 10,000 ms, TE = 69 ms, inversion time = 2500 ms, number of slices = 40, slice thickness = 3 mm, in-plane resolution = 0.86 mm × 0.86 mm) was used.

### Image analysis

To ensure sufficient data quality, all T1-weighted images were visually checked to exclude potentially artefact afflicted scans. Imaging data analysis was performed using the FMRIB’s Software Library (FSL, 5.0.9, Oxford, UK) (Smith et al. 2004). For a detailed description of processing procedures, see (Hafkemeijer et al. 2014). In short, the following pre-processing steps were conducted: brain extraction from T1-weighted images using a semi-automated tool as implemented in FSL (Smith 2002), and tissue-type segmentation into grey matter, white matter, and cerebrospinal fluid, using a voxel-based morphometric analysis (Ashburner and Friston 2000). Again, visual inspection of all grey-matter segmented images was performed to ensure data quality. The individual grey-matter images were aligned to the grey-matter MNI152 standard space (Montreal Neurological Institute, Montreal, QC, Canada) (Jenkinson et al. 2002), followed by a non-linear registration (Andersson et al. 2007). The resulting images were averaged to create a study-specific grey-matter template. Then, all native grey-matter images were non-linearly re-registered to this study-specific template and “modulated” to correct for local expansion (or contraction) due to the non-linear component of the spatial transformation. The modulated grey-matter images were then smoothed with an isotropic Gaussian kernel with a sigma of 3 mm.

The modulated images of all 257 subjects were used as a four-dimensional data set on which an independent component analysis was applied using multivariate exploratory linear optimized decomposition into independent components (Beckmann et al. 2005). This procedure decomposes the signals into spatial component maps of maximal statistical independence (Beckmann and Smith 2004). When applied on grey-matter images, this method defines fully automatically spatial components based on the covariation of grey-matter intensities among subjects (i.e., SCNs), without a predefined region of interest. As there exists no consensus on the best number of components, we restricted the number to 20. A mixture model was used to assign significance to individual voxels within a spatial map, using a standard threshold level of 0.5 (Beckmann and Smith 2004). To obtain the integrity score of the SCNs, the four-dimensional data set of grey-matter images was spatially regressed against the 20 SCN probability maps using a general linear model approach integrated in FSL (Filippini et al. 2009). This method calculates beta values (positive and negative scores) for each network. The beta scores, representing measures for the network integrity, were used in all statistical analyses. Anatomical locations were determined using the Harvard–Oxford cortical, subcortical and the cerebellar atlases implemented in FSL. An equivalent approach was used in (Hafkemeijer et al. 2014, 2016; Foster-Dingley et al. 2016).

Global brain volume, normalized for subject head size, was estimated with SIENAX (Smith et al. 2001), part of FSL (Smith et al. 2004). SIENAX starts by extracting brain and skull images from the single whole-head input data (Smith 2002). The brain image is then affine-registered to MNI152 space (Jenkinson and Smith 2001) (using the skull image to determine the registration scaling); this is primarily to obtain the volumetric scaling factor, to be used as a normalization for head size. Next, tissue-type segmentation with partial volume estimation is carried out (Zhang et al. 2001) to calculate total volume of brain tissue (including separate estimates of volumes of grey matter, white matter, peripheral grey matter, and ventricular CSF).

### Confounding variables

To adjust for potential confounders on cognition, the following variables were tested in simple regression analyses for their association with cognitive functions, and considered in the statistical models if *p* was < 0.1. Besides the variables age, sex, and education, also the variables hypertension [present yes/no, 22.6% (*n* = 58) of the subjects], lacunes [present yes/no, 10.9% (*n* = 28) of the subjects], white matter hyperintensity score (WMHs), and normalized global brain volume showed associations with memory, executive function, and fine motor skills. Visual rating of WMHs was rated according to our scheme (Fazekas et al. 1987, 1993) into absent [grade 0, 17.9% (*n* = 46) of the subjects], punctuate [grade 1, 36.1% (*n* = 93) of the subjects], early confluent [grade 2, 17.1% (*n* = 44) of the subjects], and confluent [grade 3, 10.1% (*n* = 26) of the subjects]. No association with cognition was found for diabetes [present yes/no, 4.3% (*n* = 11) of the subjects], cholesterol and smoking (yes/no, *M* = 203.7 mg/dl, SD = 41.5).

### Statistical analysis

For statistical analyses, IBM SPSS Statistics Version 22, IBM Corp., USA and R (version 3.2.4) (R-Core-Team 2016) was used. We examined the association between age and grey-matter network disintegration adjusted for sex and education in all 257 subjects (SCN (Y) ~ [age + sex + education]). We aimed to assess the predictive value of all structural covariance networks, risk factors, age-related brain abnormalities (WMH score, lacunes) as well as age, sex, and education for memory, executive function, and fine motor skills. We did not perform multiple linear regression to avoid the risks of overfitting, overadjustment, and ultimately biased estimation (Ranucci et al. 2010; Yoo et al. 2014) due to multicollinearity (intercorrelation of variables). Instead, we used random forest regression, which assesses the explanatory power of variables while accounting for all other variables. One major independent variables, even in the presence of complex interactions and multicollinearity when applying conditional inference trees (Strobl et al. 2009). We calculated 1001 conditional inference trees with unbiased variable selection using the standard parameters (5 randomly preselected variables for each split, unbiased resampling scheme) using the R package ‘party’ (version 1.0-25) (Strobl et al. 2007). From these trees, we next calculated a conditional permutation importance (following the permutation principle of the ‘mean decrease in accuracy’ importance measure) (Strobl et al. 2008) for each variable together with a 95% confidence interval from 100 repetitions. To ensure that the results were not driven by subjects with MCI, the random forest analysis was repeated excluding subjects classified as MCI.

To test if age effects on cognition or on fine motor skills are mediated by grey-matter disintegration, we applied simple mediation models for estimating indirect effect size (Hayes 2013). Mediation was evaluated separately in those grey-matter SCNs that disintegrated with aging and also showed a significant association with cognitive impairment or fine motor skills in the random forest model. In total, eight networks fulfilled these requirements. As some networks were associated with more than one cognitive domain, 25 mediation analyses were performed. Mediator effect size and 95% confidence intervals were estimated using a bootstrap-based method developed by Preacher and Hayes (Preacher and Hayes 2008). If the 95% confidence interval of the indirect effect does not contain 0, a significant mediation effect is probable, whereas no mediation is present if 0 is included in the 95% confidence interval. Mediation analyses was performed with the macro PROCESS [http://www.afhayes.com (Hayes 2013)] implemented in SPSS.

## Results

### Structural covariance networks and aging

**Fig. 2:**
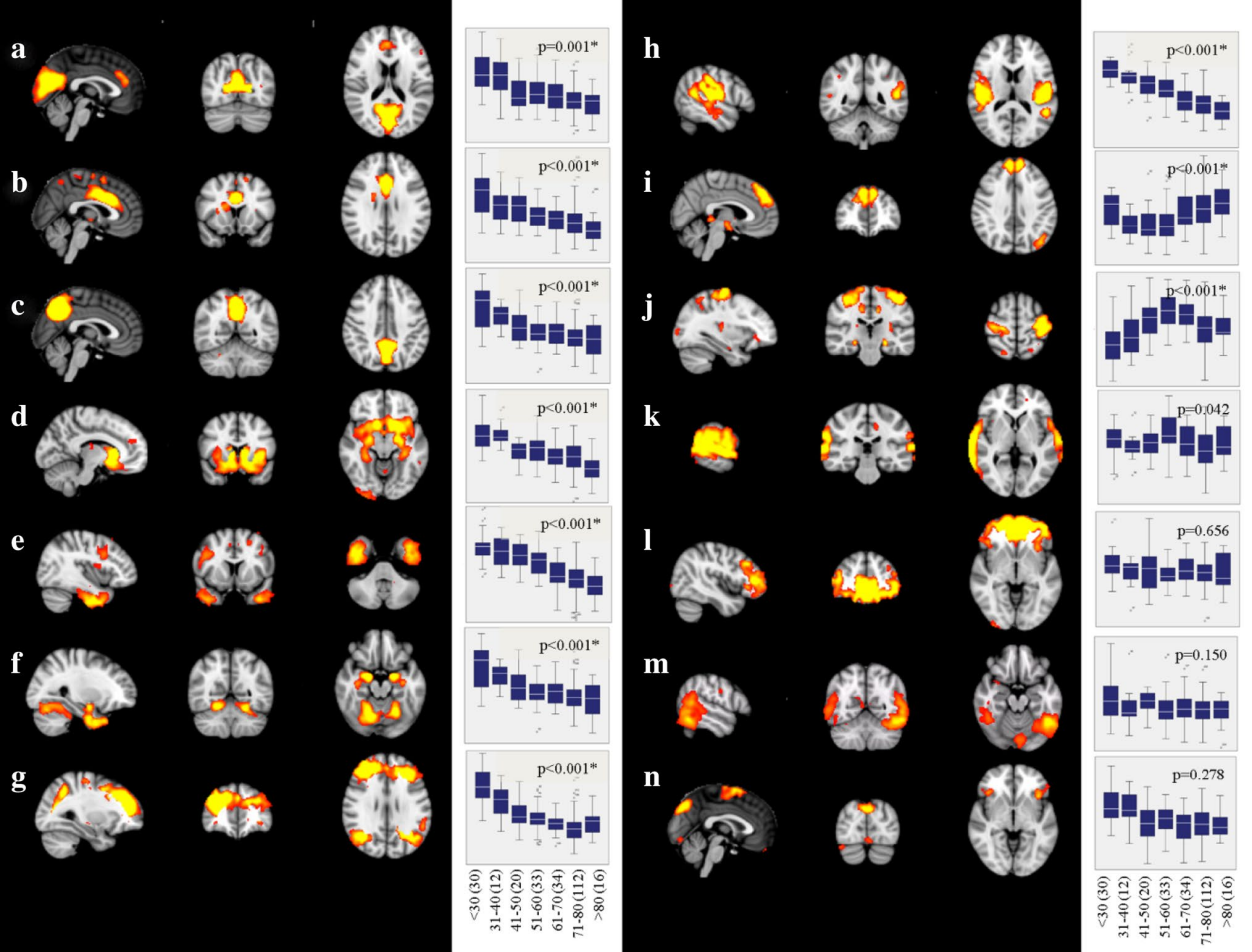
Supratentorial structural covariance networks of non-demented subjects. For areas included in each of these networks, refer to Table 1. Box–whisker plots indicate grey-matter integrity per decade, and *p* value indicates significance of age as predictor of network disintegration (adjusted for sex and education, and corrected for multiple comparisons; FDR, *q* < 0.05, significant networks are marked with an asterisk). A linear decline of grey-matter integrity with aging was found in networks (**a**–**h**), in one network (**i**) the association was u-shaped, and in another (**j**), it was inversely u-shaped. The number of subjects within the age ranges is indicated in parenthesis

**Fig. 3:**
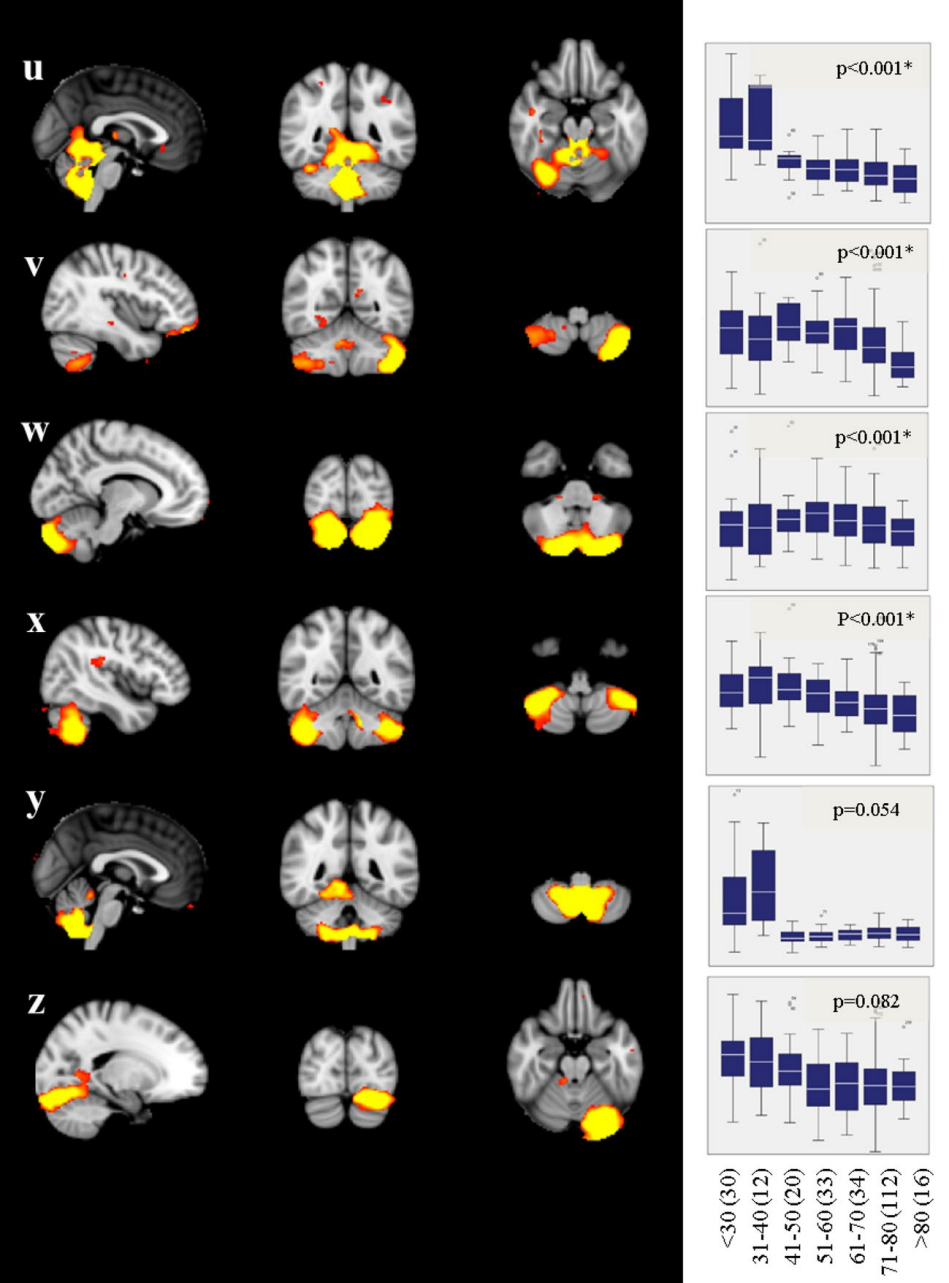
Infratentorial structural covariance networks of non-demented subjects. For areas included in each of these networks, refer to Table 2. Box–whisker plots indicate grey-matter integrity per decade, and *p* value indicates significance of age as predictor of network disintegration (adjusted for sex, and education, and corrected for multiple comparisons; FDR, *q* = 0.05, significant networks are marked with an asterisk). Networks **u**–**x** show a reduction of grey-matter network integrity with increasing age. The number of subjects within the age ranges is indicated in parenthesis

Independent component analysis revealed 14 supratentorial covariance networks (Fig. 2) and six infratentorial networks (Fig. 3) to be present in community-dwelling subjects free of stroke and dementia. The areas included in each of the supratentorial networks are described in Table 1. Those of the infratentorial networks are described in Table 2.

**Table 1:** Areas included in supratentorial structural covariance networks (cluster size *k* > 1000 voxels)

SCN	Voxels	MNI coordinates			Location	hem.
		*x*	*y*	*z*		
a	9488	2	− 70	22	Cuneal cortex	R
	1682	2	32	30	Paracingulate gyrus	R
b	7872	0	16	28	Cingulate gyrus, anterior division	R
	1683	− 34	− 62	− 4	Occipital fusiform gyrus	L
	1139	− 34	− 6	2	Insular cortex	L
c	6392	− 2	− 62	28	Precuneus cortex	L
	1095	12	− 66	− 26	Cerebellum (VI)	R
d	15,580	10	14	− 12	Subcallosal cortex	R
	1484	28	− 100	8	Occipital pole	R
e	10,442	40	− 4	− 40	Inferior temporal gyrus, anterior division	R
	5355	− 38	8	− 38	Temporal pole	L
	1466	0	20	40	Paracingulate gyrus	R
	1313	10	38	− 4	Cingulate gyrus, anterior division	R
	1106	− 32	22	− 4	Insular cortex	L
f	10,911	18	− 4	− 20	Amygdala	R
	3841	− 20	− 4	− 22	Amygdala	L
	1036	42	− 22	56	Postcentral gyrus	R
g	17,295	30	46	20	Frontal pole	R
	3753	− 42	− 72	24	Lateral occipital cortex, superior division	L
	3366	34	− 66	20	Lateral occipital cortex, superior division	R
h	9650	− 48	− 26	16	Parietal operculum cortex	L
	7478	50	− 28	16	Parietal operculum cortex	R
	1028	10	− 26	34	Cingulate gyrus, posterior division	R
i	5332	8	50	34	Superior frontal gyrus	R
	4809	− 8	− 32	0	Thalamus	L
	1958	− 42	− 66	40	Lateral occipital cortex, superior division	L
j	6772	36	− 24	56	Postcentral gyrus	R
	5894	− 40	− 18	58	Precentral gyrus	L
	1687	30	− 56	− 32	Cerebellum (VI)	R
	1183	24	− 34	− 2	Hippocampus	R
	1016	− 24	− 22	− 12	Hippocampus	L
k	8071	66	− 34	10	Superior temporal gyrus	R
	5038	− 70	− 26	8	Superior temporal gyrus	L
	2810	− 54	− 70	− 36	Cerebellum (crus I)	L
	2500	− 14	36	22	Paracingulate gyrus	L
l	23,648	2	44	− 10	Paracingulate gyrus	R
	1581	34	− 98	− 2	Occipital pole	R
m	11,130	− 48	− 54	− 18	Inferior temporal gyrus, temporooccipital part	L
	5891	44	− 40	− 32	Cerebellum (crus I)	R
n	4451	2	2	66	Supplementary motor cortex	R
	4433	− 38	22	− 2	Insular cortex	L
	2532	2	− 76	40	Precuneus cortex	R
	1843	− 40	− 58	− 22	Temporal occipital fusiform cortex	L
	1115	40	− 72	− 20	Cerebellum (crus I)	R

*SCN* structural covariance network (referring to Fig. 2), *hem*. hemisphere

**Table 2:** Areas included in infratentorial structural covariance networks (cluster size *k* > 1000 voxels)

SCN	Voxels	MNI coordinates			Location	hem.
		*x*	*y*	*z*		
u	15,844	2	− 48	− 50	Cerebellum (right IX)	R
	1247	12	− 38	48	Precuneus	R
v	4741	− 42	− 72	− 44	Cerebellum (crus II)	L
	2314	4	52	− 28	Frontal medial cortex	R
	2022	44	− 58	− 52	Cerebellum (VIIb)	R
w	9773	− 26	− 88	− 34	Cerebellum (crus II)	L
x	15,801	42	− 54	− 42	Cerebellum (crus I)	R
y	4618	10	− 58	− 54	Cerebellum (right IX)	R
	2411	12	− 42	− 16	Cerebellum (right I–IV)	R
z	6105	− 28	− 78	− 22	Cerebellum (crus I)	L
	2357	16	− 106	0	Occipital pole	R

*SCN* structural covariance network (referring to Fig. 3), *hem*. hemisphere

Ten supratentorial (Fig. 2a–j) and four infratentorial networks (Fig. 3u–x) lost integrity with advancing age. The association with aging was linear in all but two networks (Fig. 2i, j).

### Structural covariance network disintegration and cognition

**Fig. 4:**
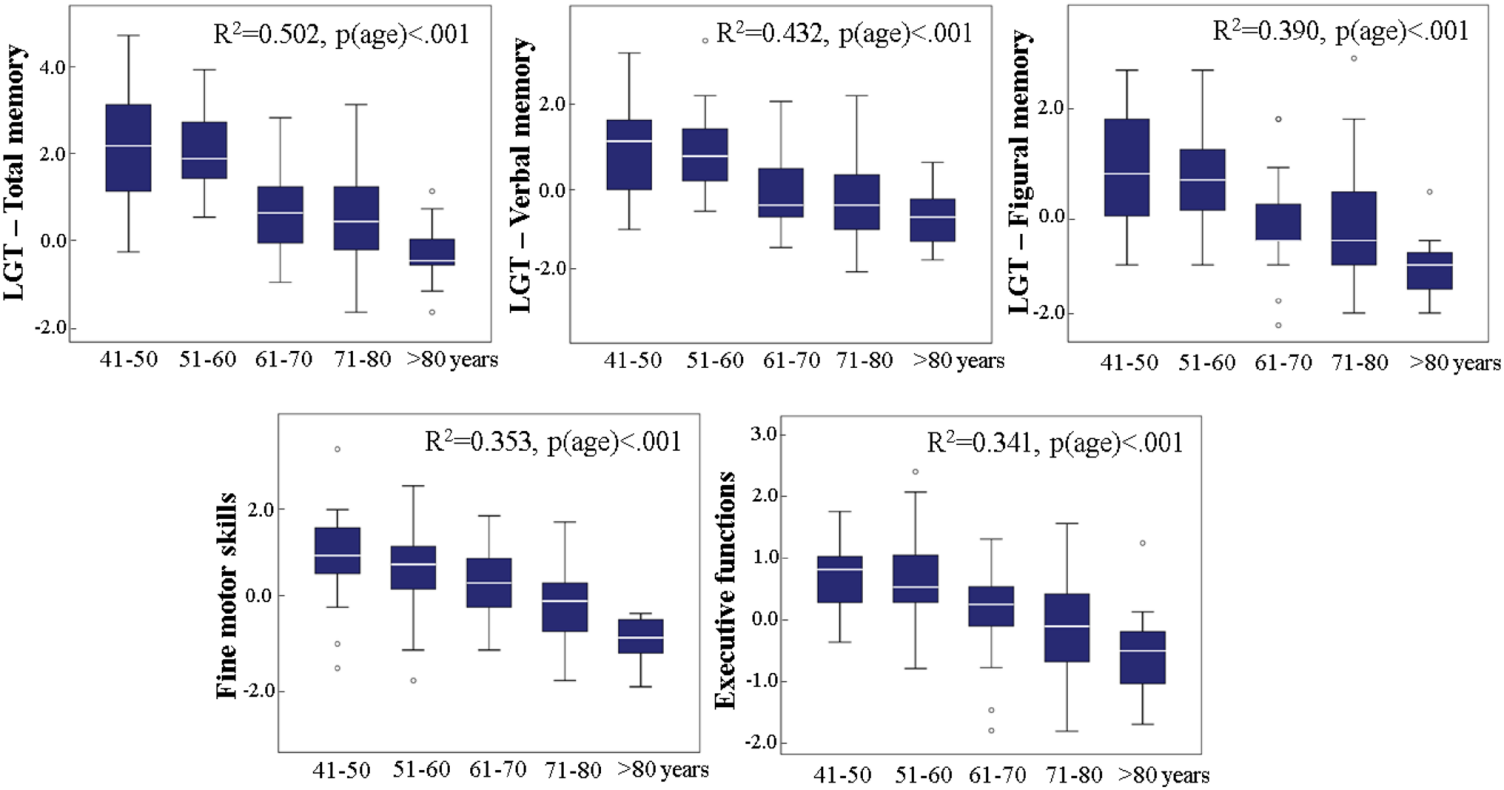
Cognitive performance changes with aging per decade in memory, executive functions, and fine motor skills. All *R*^2^’s are based on a hierarchical linear regression (step 1: sex, education; step 2: age)

As can be seen from Fig. 4, in our study, population advancing age was associated with performance decrease in all cognitive domains.

**Fig. 5:**
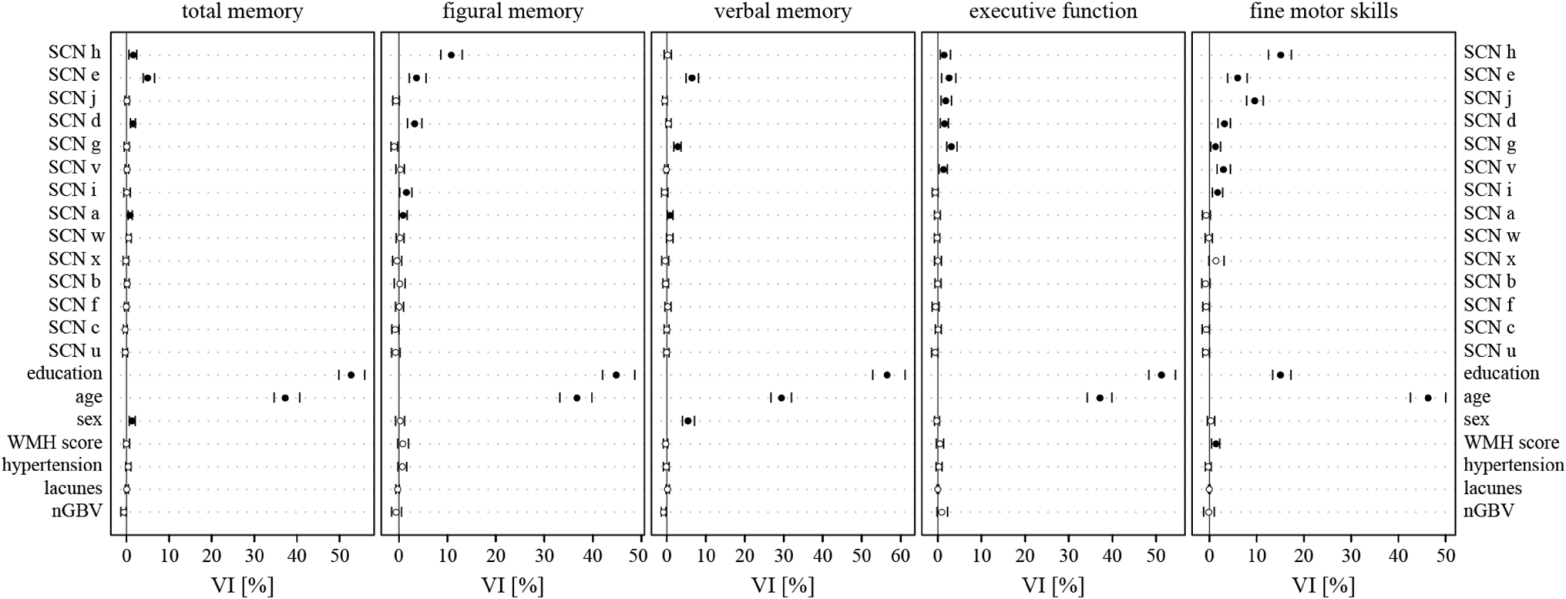
Descriptive ranking of variable importance (VI) for the prediction of cognitive and motor skills, as determined by random forest regression. Only networks changing integrity with age were included in the model. In addition, demographical variables (education, age, and sex), age-related brain abnormalities (WMH score, lacunes), normalized global brain volumes (nGBV), and hypertension were included. Spheres and vertical lines indicate VI mean and 95% confidence interval. Only if the confidence interval did not overlap with zero, a variable was considered to have a significant contribution in predicting the performance (filled dot). Education and age showed the highest variable importance in all domains. Additional association with the domains were found for the secondary somatosensory network (**h**), the temporal network (**e**), the sensorimotor network (**j**), the limbic network (**d**), the fronto-parietal network (**g**), a cerebellar network (**v**), the fronto-occipital network (**i**), and the cuneal network (**a**)

Correlation matrices of the structural covariance networks revealed a high intercorrelation among the networks (data not shown). Therefore, to assess the contribution of each network to memory, executive function, and fine motor skills, while accounting for intercorrelations (multicollinearity), we applied random forest regression and calculated the conditional variable importance (VI). Education and age had the highest variable importance (Fig. 5; Table 3). SCNs which contributed independently to memory were the secondary somatosensory network (Fig. 2h), the temporal network (Fig. 2e), the limbic network (Fig. 2d), the fronto-parietal network (Fig. 2g), the fronto-occipital network (Fig. 2i), and the cuneal network (Figs. 2a, 5; Table 3). Those contributing to executive function were the secondary somatosensory network (Fig. 2h), the temporal network (Fig. 2e), the sensorimotor network (Fig. 2j), the limbic network (Fig. 2d), the fronto-parietal network (Fig. 2g), and a cerebellar network (Figs. 3v, 5; Table 3). The secondary somatosensory network (Fig. 2h), the temporal network (Fig. 2e), the sensorimotor network (Fig. 2j), the limbic network (Fig. 2d), the fronto-parietal network (Fig. 2g), a cerebellar network (Fig. 3v), and the fronto-occipital network contributed independently to fine motor skills of study participants (Figs. 2i, 5; Table 3). The WMH score had predictive value only for fine motor skills. Normalized global brain volume, hypertension, and lacunes were not associated with any outcome (Fig. 5).

**Table 3:** Conditional variable importance mean (point estimator) and 95% confidence interval of structural covariance networks, age-related brain abnormalities, risk factors and demographics for cognitive outcomes and fine motor skills related to Fig. 5

	Total memory	Figural memory	Verbal memory	Executive function	Fine motor skills
SCN h	1.56 (0.51; 2.35)	10.8 (8.64; 13.04)	0.23 (− 0.7; 1.14)	1.47 (0.56; 2.87)	15.08 (12.45; 17.35)
SCN e	4.95 (3.89; 6.51)	3.64 (2.12; 5.59)	6.44 (4.89; 8.12)	2.61 (0.91; 4.1)	5.99 (3.82; 7.93)
SCN j	0.12 (− 0.37; 0.56)	− 0.61 (− 1.29; 0.03)	− 0.54 (− 1.11; 0.03)	1.85 (0.77; 3.14)	9.6 (7.87; 11.34)
SCN d	1.47 (0.91; 2.05)	3.24 (1.76; 4.73)	0.43 (− 0.18; 1.03)	1.58 (0.57; 2.42)	3.2 (1.82; 4.41)
SCN g	0.07 (− 0.56; 0.56)	− 0.98 (− 1.63; − 0.3)	2.82 (1.76; 3.66)	3.11 (2.04; 4.39)	1.29 (0.25; 2.35)
SCN v	0.12 (− 0.33; 0.48)	0.33 (− 0.64; 1.13)	− 0.13 (− 0.53; 0.28)	1.35 (0.32; 2.17)	2.96 (1.61; 4.39)
SCN i	0.08 (− 0.65; 0.85)	1.56 (0.13; 2.64)	− 0.56 (− 1.4; 0.25)	− 0.59 (− 1.27; 0.06)	1.73 (0.59; 2.81)
SCN a	0.81 (0.35; 1.32)	0.87 (0.01; 1.69)	0.87 (0.14; 1.53)	− 0.12 (− 0.74; 0.57)	− 0.7 (− 1.49; 0.13)
SCN w	0.51 (− 0.04; 1.04)	0.25 (− 0.62; 1.05)	0.73 (− 0.1; 1.56)	− 0.17 (− 0.82; 0.45)	− 0.11 (− 0.93; 0.55)
SCN x	− 0.15 (− 0.84; 0.45)	− 0.42 (− 1.31; 0.57)	− 0.41 (− 1.34; 0.45)	0.01 (− 0.75; 0.83)	1.39 (− 0.14; 3.06)
SCN b	0.14 (− 0.42; 0.57)	0.19 (− 1.03; 1.27)	− 0.28 (− 1.01; 0.41)	0.01 (− 0.67; 0.75)	− 0.83 (− 1.61; 0.09)
SCN f	− 0.05 (− 0.49; 0.4)	0.06 (− 0.73; 0.96)	0.25 (− 0.51; 1.02)	− 0.51 (− 1.29; 0.31)	− 0.69 (− 1.41; − 0.02)
SCN c	− 0.34 (− 0.75; 0.11)	− 0.77 (− 1.51; − 0.04)	0 (− 0.68; 0.54)	0.11 (− 0.59; 0.78)	− 0.69 (− 1.55; − 0.02)
SCN u	− 0.3 (− 0.97; 0.19)	− 0.69 (− 1.53; 0.18)	− 0.05 (− 0.76; 0.56)	− 0.62 (− 1.43; 0.01)	− 0.79 (− 1.41; 0)
Education	52.7 (49.81; 55.84)	44.82 (41.97; 48.65)	56.51 (52.79; 61.11)	51.22 (48.36; 54.42)	15.02 (13.37; 17.24)
Age	37.24 (34.57; 40.6)	36.72 (33.18; 39.78)	29.42 (26.7; 31.95)	37.18 (34.2; 39.83)	46.29 (42.53; 50)
Sex	1.27 (0.61; 1.99)	0.29 (− 0.72; 1.17)	5.43 (3.98; 7.01)	− 0.25 (− 0.76; 0.4)	0.31 (− 0.48; 1.05)
WMH score	− 0.04 (− 0.66; 0.68)	0.81 (− 0.29; 1.98)	− 0.29 (− 0.91; 0.21)	0.53 (− 0.35; 1.34)	1.38 (0.4; 2.17)
Hypertension	0.41 (− 0.19; 0.98)	0.71 (− 0.24; 1.62)	− 0.15 (− 0.93; 0.53)	0.3 (− 0.39; 0.98)	− 0.25 (− 0.87; 0.33)
Lacunes	0.06 (− 0.28; 0.41)	− 0.26 (− 0.63; 0.03)	0.18 (− 0.2; 0.59)	0.02 (− 0.16; 0.21)	− 0.04 (− 0.18; 0.1)
nGBV	− 0.63 (− 1.38; − 0.1)	− 0.59 (− 1.53; 0.52)	− 0.88 (− 1.45; − 0.28)	0.93 (− 0.19; 2.22)	− 0.15 (− 1.27; 0.98)

By means of MMSE norm scores, 34 subjects were classified as being mild cognitively impaired. Repetition of the random forest analyses without these subjects revealed almost unchanged results. Equivalently to the above results, age, sex, and education had the utmost association with cognitive and motor domains. Similar, memory was associated with the limbic network (Fig. 2d), the temporal network (Fig. 2e), the fronto-parietal network (Fig. 2g), the secondary somatosensory network (Fig. 2h), and additionally two cerebellar networks (Fig. 3v, w). SCNs contributing to executive functions were the temporal network (Fig. 2e), the sensorimotor network (Fig. 2j), the limbic network (Fig. 2d), the fronto-parietal network (Fig. 2g), and a cerebellar network (Fig. 3v). The normalized global brain volume and all but the fronto-occipital network of the total sample were associated with fine motor skills.

### Mediation analyses

Mediation analyses were performed for each SCN disintegrating with age and showing an association with cognition or fine motor skills. Of 25 mediation analyses, in 13 cases, the effect of age on cognition or fine motor skills was mediated by an SCN. Seven of the tested SCNs were among these mediators (Table 4). A negative association of age on cognition and fine motor skills, ranging between − 0.1612 and − 0.5196 (total effect), was found. The total effect gets reduced by the indirect effect [− 0.0030, 0.0206] of the mediator, i.e., the SCN, reducing the effect of age on cognition and fine motor skills [direct effect, − 0.0275, − 0.4813]. The ratio of the indirect to the total effect ranges between 7.38 and 23.48%.

**Table 4:** Mediation models assessing the effect of grey-matter disintegration as measured by the SCNs on the relationship between age and cognitive performance and fine motor skills

	*N*	Total effect (*c*)	Direct effect (*c*′)	Indirect effect (*ab*)	Ratio^a^	Bootstrapped CI
Total memory						
Temporal network (e)	206	− 0.5196	− 0.4237	− 0.0959	0.1845	**− 0.1800, − 0.0279**
Secondary somatosensory network (h)	206	− 0.5196	− 0.4753	− 0.0443	0.0852	**−** 0.1264, 0.0315
Limbic network (d)	206	− 0.5196	− 0.4813	− 0.0383	0.0738	**− 0.0867, − 0.0056**
Cuneal network (a)	206	− 0.5196	− 0.5461	− 0.0265	**−** 0.0509	**−** 0.0032, 0.0751
Figural memory						
Secondary somatosensory network (h)	206	− 0.1612	− 0.1234	− 0.0378	0.2348	**− 0.0741, − 0.0103**
Temporal network (e)	206	− 0.1612	− 0.1374	− 0.0238	0.1477	**−** 0.0513, 0.009
Limbic network (d)	206	− 0.1612	− 0.1459	− 0.0153	0.1048	**− 0.0346, − 0.0020**
Fronto-occipital network (i)	206	− 0.1612	− 0.1543	− 0.0069	0.0426	**−** 0.0281, 0.0134
Cuneal network (a)	206	− 0.1612	− 0.1693	− 0.0081	**−** 0.0505	− 0.00046, 0.0307
Verbal memory						
Temporal network (e)	206	− 0.2474	− 0.1953	− 0.0521	0.2108	**− 0.0996, − 0.0103**
Fronto-parietal network (g)	205	− 0.2433	− 0.2268	− 0.0164	0.0675	**−** 0.0409, 0.0010
Cuneal network (a)	206	− 0.2474	− 0.2680	0.0206	na	**0.0018, 0.0532**
Executive functions						
Fronto-parietal network (g)	213	− 0.0311	− 0.0275	− 0.0036	0.1144	**− 0.0074, − 0.0014**
Temporal network (e)	214	− 0.0319	0.0270	− 0.0048	0.1520	**−** 0.0111, 0.0005
Sensorimotor network (j)	214	− 0.0319	− 0.0297	− 0.0022	0.0690	**−** 0.0061, 0.0008
Limbic network (d)	214	− 0.0319	− 0.0288	− 0.0030	0.0955	**− 0.0069, − 0.0001**
Secondary somatosensory network (h)	214	− 0.0319	− 0.0300	− 0.0019	0.0598	**−** 0.0076, 0.0035
Cerebellar network (v)	214	− 0.0319	− 0.0295	− 0.0024	0.0762	**−** 0.0063, 0.0005
Fine motor skills						
Secondary somatosensory network (h)	212	− 0.3034	− 0.2446	− 0.0588	0.1938	**− 0.1172, − 0.0115**
Sensorimotor network (j)	212	− 0.3034	− 0.2631	− 0.0403	0.1328	**− 0.0763, − 0.0161**
Temporal network (e)	212	− 0.3034	− 0.2450	− 0.0583	0.1923	**− 0.1061, − 0.0151**
Limbic network (d)	212	− 0.3034	− 0.2685	− 0.0348	0.1148	**− 0.0684, − 0.0122**
Cerebellar network (v)	212	− 0.3034	− 0.2745	− 0.0289	0.0951	**− 0.0613, − 0.0056**
Fronto-occipital network (i)	212	− 0.3034	− 0.2794	− 0.0240	0.0790	**−** 0.0580, 0.0004
Fronto-parietal network (g)	211	− 0.2982	− 0.2779	− 0.0204	0.0683	**−** 0.0576, 0.0028

*Dependent variables*: total memory, figural memory, verbal memory, executive functions, fine motor skills; *independent variable*: age; *mediator*: structural covariance networks. All models are adjusted for sex and education

Significant results are highlighted in bold

*CI* confidence interval, *na* not applicable

^a^Ratio of indirect effect to total effect of independent variable on dependent variable, i.e., the amount the mediator can account for of the total effect. Because of missing data, effective sample size varied between 206 and 214

## Discussion

In this study, we identified structural covariance networks that disintegrate with increasing age and related them to cognitive function independently of risk factors and age-related brain abnormalities including vascular lesions and brain atrophy. Of 14 networks that lost integrity with aging, eight were related to either cognitive or motor function, of which seven mediated the effect between age and cognition and motor function. With the exception of two networks, the loss of network integrity was linear with advancing age. Besides age and education, disintegration of the temporal, limbic, fronto-parietal, fronto-occipital, sensorimotor, secondary somatosensory, cuneal and a cerebellar network showed strongest association with cognitive or motor function. Importantly, these results remained largely unchanged when excluding subjects with Mild Cognitive Impairment (MCI) potentially showing incipient neuropathological alterations. The grey-matter networks identified in our investigation showed an overlap with covariance networks described by prior literature (Hafkemeijer et al. 2014, 2016), and also partly overlapped with intrinsic functional networks identified with BOLD-contrast imaging (Smith et al. 2009). This is not implausible, since direct anatomical connections between areas are related to functional connectivity but not mandatory, i.e., functional connectivity can arise in the absence of structural connectivity (Alexander-Bloch et al. 2013). Moreover, the results of the mediation analyses confirmed the partial mediating role of grey-matter disintegration in the relation between age and cognition and fine motor skills, suggesting a neuronal basis accounting for at least part of the association between age and cognition and fine motor skills. The mediator could account for up to roughly 23% of the total effect (Table 4, ratio). The results of the current study are in line with the view that growth and degeneration of the cortex occurs at the level of networks, rather than in a region specific manner (Alexander-Bloch et al. 2013; Hafkemeijer et al. 2014). According to our data network disintegration is seen already above the age of 30, a finding in keeping with previous literature reporting grey-matter loss soon after adolescence (Courchesne et al. 2000; Giedd 2004; Alexander-Bloch et al. 2013).

There exists little information on age-related cortical network degeneration so far. Previous investigations used subprofile scaling models (Brickman et al. 2007; Steffener et al. 2013) or partial least squares (DuPre and Spreng 2017) and reported that networks that best differentiate between younger and older subjects were neurocognitive networks linked to attention (Brickman et al. 2007; Steffener et al. 2013), language (Brickman et al. 2007), memory (Brickman et al. 2007; Steffener et al. 2013), executive functions (Brickman et al. 2007), and fluid abilities (Steffener et al. 2013). In our study, on top of age, sex, and education, the grey-matter covariance networks presented up to 10.08% of importance for memory, 3.11% for executive function, and 15.08% for fine motor skills. Normalized global brain volume, lacunes, and hypertension did show an association with cognitive and motor abilities, and WMH score was associated with fine motor skills only. Of the eight networks that were identified to be important for memory, executive function, and fine motor skill in the random forest analyses, seven networks revealed a partly mediating effect between age and cognitive and motor outcome. Hence, the disintegration of grey-matter covariance networks constitutes an additional potential factor for cognitive decline in the aging brain independent of normalized global brain volume or other confounders. Determining the causes for this selective vulnerability of disintegration of certain networks with aging leads to the developmental and maturing processes underlying these covariance networks. Coordinated neurodevelopment has been suggested to constitute the basis of morphological covariance which may be induced in different ways. Provisional theories suggest that the correlation of phenotypic traits could be evoked by shared genetic influences, common environmental factors, inductive signaling from one developing tissue to another or simultaneous exposure to signals from third party, timing of development or the sharing of a developmental precursor (Riska 1986; Alexander-Bloch et al. 2013). Hence, coordinated neurodevelopment might form the scaffold of structural covariance. Preliminary imaging results underpin this hypothesis. A strong covariance has been identified for growth and disintegration in volume of networks (DuPre and Spreng 2017). In a longitudinal study, it was shown that areas showing structural covariance were also correlated in their rate of change considering cortical thickness (Alexander-Bloch et al. 2013).

Increasing age differentially affected the integrity of networks determined in our study. While most networks showed a linear disintegration with age, some remained stable over the entire age range between 20 and 87 years, and two had an u-shaped trajectory. Stable networks were found to connect the superior temporal gyri, the cerebellum and the hippocampus (Fig. 2k), frontal areas with the occipital pole (Fig. 2l), the inferior temporal gyrus and the cerebellum (Fig. 2m) and the supplementary motor area with the insular, the precuneus, the temporal occipital fusiform cortex, and the cerebellum (Fig. 2n). Networks with decreasing connectivity included a cuneal network, mainly showing covariance between the cuneus and the paracingulate gyrus, an anterior cingulate network, with associations between the anterior cingulate cortex, the occipital fusiform gyrus and the insular cortex, a precuneal network, showing associations between the precuneus and the cerebellum, a limbic network, connecting subcortical and cortical areas, a temporal network, showing major associations between the inferior temporal gyrus, the temporal pole, and the paracingulate gyrus, a subcortical network, mainly comprising the amygdala, a fronto-parietal network, connecting the frontal poles with the lateral occipital cortices, a secondary sensorimotor network, including both parietal opercular cortices and the posterior cingulate gyrus (Fig. 2a–h), and finally four cerebellar networks (Fig. 3u–x). Notably, two networks comprising the superior frontal gyrus and the thalamus (Fig. 2i) and another showing connection between the pre- and postcentral gyri, the cerebellum, and the hippocampi (Fig. 2j) showed (inverted) u-shaped trajectories. While the inverted u-shape in network j, which is primarily a sensorimotor network, indicates that increasing network integration in this important functional network may occur up to the age of 60, it is difficult to explain the u-shaped trajectory of network i. We cannot exclude that this was a chance finding, although all statistics in our investigation were corrected for multiple comparisons. It needs to be emphasized that the results of current cross-sectional investigation need to be replicated by longitudinal studies which ideally follow the same individuals over their life span.

Our results revealed an association between network disintegration and higher order cognitive functions and fine motor skills, respectively. Memory has been associated with the grey-matter integrity of the temporal, secondary somatosensory, limbic, cuneal and the fronto-occipital network. The strongest association with executive function was seen for the fronto-parietal, temporal, sensorimotor, limbic, secondary somatosensory, and the cerebellar network. The sensorimotor, secondary somatosensory, temporal, limbic, fronto-occipital, fronto-parietal, and cerebellar network were identified as being the most important determinants for fine motor skills. The temporal, limbic, fronto-occipital, and the secondary somatosensory network have been associated with more than one cognitive domain or fine motor skill, respectively.

Four cerebellar networks showed an association with age, but only one with fine motor skills. This was somewhat surprising, since the cerebellum has repeatedly been shown to be involved in cognitive and motor function (Stoodley 2012). In addition, this is in contrast to a prior study on subjects > 75 years reporting no association between cerebellar grey-matter networks and age, but an association with psychomotor speed (Hafkemeijer et al. 2014; Foster-Dingley et al. 2016). An explanation for this discrepancy might be found in our networks which encompass widespread cerebellar regions instead of rather small circumscribed areas. A prior study using functional imaging revealed a specialization of cerebellar subfields depending on the task performed (Stoodley et al. 2011) highlighting narrow circumscribed cerebellar areas. It is, therefore, thinkable that our networks over-represent a specific domain.

We here examined grey-matter network changes in a cross-sectional design. This approach does not allow inferences on intra-individual trajectories of grey-matter network disintegration over time limiting the interpretability of individual association between risk factors, network disintegration, and the effect on cognition. Longitudinal designs are considered optimal to examine intra-individual changes, but imaging studies spanning long time periods also face problems such as advancements in sequence technology or even hardware changes hampering comparability.

The inclusion of individuals between 20 and 38 years of age allowed to broaden the age range of our study and to assess covariance of networks from post-adolescence up to the ninth decade of life. However, these subjects have not been cognitively tested and led to a three-modal distribution of age with an underrepresentation of subjects in the age range between 35 and 45 years. This group of subjects was added from a different study with the same imaging protocol, but without neuropsychological assessment. We are aware of this drawback, but inclusion of this group of individuals allowed to broaden the age range of our study and to assess covariance of networks from post-adolescence up to the ninth decade of life. Third, the number of independent components was chosen arbitrarily. In functional imaging, most frequently, a number between 10 and 20 are chosen (Barkhof et al. 2014). To obtain a better sub-network segmentation, we chose a higher number. Finally, the mediation analyses were not corrected for multiple comparisons as with using confidence intervals for significant result interpretation, and no *p* values are calculated by the software PROCESS by Andrew Hayes. Hence, we assumed that the null is always true, and therefore, the probability of not making any type I error would be 0.95^13^ = 0.51, which means that 1 of the 13 analyses has a probability of 0.49 of being a false positive. Thus, one of the significant 13 results of the mediation analyses has a 49% chance of being incorrect.

Our study cannot determine whether the observed association between network disintegration and cognitive impairment with advancing age is at least partly due to evolving neurodegenerative disease. Future longitudinal studies will have to determine if any of the identified covariance networks have the prognostic potential to identify individuals with a high risk for developing mild cognitive impairment or conversion to dementia beyond what can be expected for the measurement of regional brain atrophy alone. They will also have to determine as to how the prognostic value of structural covariance networks compares to other dementia biomarkers such as CSF amyloid and tau or amyloid PET.
